# Pleiotropic Effects of Bitter Taste Receptors on [Ca^2+^]_i_ Mobilization, Hyperpolarization, and Relaxation of Human Airway Smooth Muscle Cells

**DOI:** 10.1371/journal.pone.0131582

**Published:** 2015-06-29

**Authors:** Blanca Camoretti-Mercado, Susan H. Pauer, Hwan Mee Yong, Dan’elle C. Smith, Deepak A. Deshpande, Steven S. An, Stephen B. Liggett

**Affiliations:** 1 Department of Medicine and the Center for Personalized Medicine and Genomics, University of South Florida Morsani College of Medicine, Tampa, FL, United States of America; 2 Department of Environmental Health Sciences, Johns Hopkins Bloomberg School of Public Health, Baltimore, MD, United States of America; 3 Department of Medicine and Center for Translational Medicine, Thomas Jefferson University, Philadelphia, PA, United States of America; 4 Department of Molecular Pharmacology and Physiology, University of South Florida Morsani College of Medicine, Tampa, FL, United States of America; University of North Dakota, UNITED STATES

## Abstract

Asthma is characterized by airway inflammation and airflow obstruction from human airway smooth muscle (HASM) constriction due to increased local bronchoconstrictive substances. We have recently found bitter taste receptors (TAS2Rs) on HASM, which increase [Ca^2+^]_i_ and relax the muscle. We report here that some, but not all, TAS2R agonists *decrease* [Ca^2+^]_i_ and relax HASM contracted by G-protein coupled receptors (GPCRs) that stimulate [Ca^2+^]_i_. This suggests both a second pathway by which TAS2Rs relax, and, a heterogeneity of the response phenotype. We utilized eight TAS2R agonists and five procontractile GPCR agonists in cultured HASM cells. We find that heterogeneity in the inhibitory response hinges on which procontractile GPCR is activated. For example, chloroquine inhibits [Ca^2+^]_i_ increases from histamine, but failed to inhibit [Ca^2+^]_i_ increases from endothelin-1. Conversely, aristolochic acid inhibited [Ca^2+^]_i_ increases from endothelin-1 but not histamine. Other dichotomous responses were found when [Ca^2+^]_i_ was stimulated by bradykinin, angiotensin, and acetylcholine. There was no association between [Ca^2+^]_i_ inhibition and TAS2R subtype, nor whether [Ca^2+^]_i_ was increased by G_q_- or G_i_-coupled GPCRs. Selected studies revealed a correlation between [Ca^2+^]_i_ inhibition and HASM cell-membrane hyperpolarization. To demonstrate physiologic correlates, ferromagnetic beads were attached to HASM cells and cell stiffness measured by magnetic twisting cytometry. Consistent with the [Ca^2+^]_i_ inhibition results, chloroquine abolished the cell stiffening response (contraction) evoked by histamine but not by endothelin-1, while aristolochic acid inhibited cell stiffening from endothelin-1, but not from histamine. In studies using intact human bronchi, these same differential responses were found. Those TAS2R agonists that decreased [Ca^2+^]_i_, promoted hyperpolarization, and decreased HASM stiffness, caused relaxation of human airways. Thus TAS2Rs relax HASM in two ways: a low-efficiency *de novo* [Ca^2+^]_i_ stimulation, and, a high-efficiency inhibition of GPCR-stimulated [Ca^2+^]_i_. Furthermore, there is an interaction between TAS2Rs and some GPCRs that facilitates this [Ca^2+^]_i_ inhibition limb.

## Introduction

Asthma is a disease characterized by airway inflammation and airflow limitation caused by contraction of airway smooth muscle (ASM). Contraction of ASM is due to local accumulation of agonists such as acetylcholine (Ach) and histamine, which activate G-protein coupled receptors (GPCRs) on ASM [1,2]. Indeed, the bronchoconstrictive GPCRs all increase [Ca^2+^]_i_ via coupling to G_αq_, or less commonly, G_αi_ [1]. Thus a number of GPCR antagonists acting at these receptors are used for treating asthma, and are considered “indirect” bronchodilators. The only class of direct bronchodilators is composed of agonists for ASM β_2_-adrenergic receptors (β_2_ARs), which couple to G_αs_, increase cAMP, and relax ASM through a series of events mediated by protein kinase A. The use of β-agonists, however, is associated with tachyphylaxis (tolerance) [3], increased bronchial hyperresponsiveness [4,5], interindividual variability [6], and worsening asthma and mortality [7–9].

These issues have led to our search for other drug targets that promote human ASM (HASM) relaxation [10]. We found that bitter taste receptors (TAS2Rs) are expressed on HASM cells, and when activated cause marked relaxation [11,12]. These findings have been corroborated by several other groups [13–16] although there remains some debate over the mechanism of action. TAS2Rs are broadly tuned receptors that display relatively low apparent affinities (μM to mM range) for the vast majority of currently recognized agonists [17]. In pharmacological studies in HASM using agonists for the most highly expressed TAS2R subtypes, we demonstrated that TAS2R stimulated [Ca^2+^]_i_ mobilization [11]. Intracellular cAMP levels remained unchanged in HASM exposed to TAS2R agonists [11]. This signaling is consistent with the pathway described for TAS2R in taste cells, where TAS2R couple to gustducin, and its βγ subunit activates phospholipase C, generating inositol 1,4,5-trisphosphate (IP_3_). IP_3_ acting on its receptor releases Ca^2+^ from the endoplasmic reticulum, and in taste cells this leads to release of neurotransmitter, activation of a transient receptor potential (TRP) channel, and depolarization of the cell membrane [18]. Such depolarization in the ASM cell would be expected to cause ASM contraction. However, TAS2R agonists relax ASM, and in fact cause hyperpolarization of the membrane [11], and thus the signaling of TAS2R in ASM diverges from that observed in taste cells [19,20]. Of note, TAS2R agonists cause membrane hyperpolarization and ASM relaxation of isolated cells as well as intact airways at baseline, i.e., in the absence of any procontractile stimulus [11]. However, the majority of physiological studies that we [11,12,21–24] and others [14–16] have performed with human, nonhuman primate, mouse, or guinea pig have been under circumstances where the muscle is contracted with receptor agonists such as Ach and histamine. These conditions more closely resemble the pathogenic settings of airflow obstruction in asthma. These spasmogens act at their cognate receptors to also increase [Ca^2+^]_i_ which ultimately activates myosin light chain kinase which phosphorylates regulatory light chains of myosin and evokes cross-bridge cycling and tension generation [25,26]. We have shown that the increase in [Ca^2+^]_i_ caused by TAS2Rs, though, appears to be compartmentalized, which may be the basis for its relaxation effect, as compared to G_q_/G_i_-coupled receptors which evoke a more global increase in [Ca^2+^]_i_ and contract ASM [11]. In studies of mouse ASM cells, it has recently been shown that TAS2Rs can also inhibit [Ca^2+^]_i_ that has been elevated by procontractile GPCR agonists [15]. We hypothesized that TAS2Rs act to inhibit [Ca^2+^]_i_ that has been stimulated by GPCRs, and that this response requires lower concentrations of TAS2R agonist compared to the low-efficiency [Ca^2+^]_i_ stimulatory pathway. We proposed that upon activation with contractile GPCR agonists, the TAS2R signaling from this pathway will antagonize cell membrane depolarization, promote a decrease in single cell stiffness, and cause relaxation of human airways. Based on the heterogeneity of the [Ca^2+^]_i_-inhibition response observed in initial studies, we further hypothesized that the capacity for TAS2Rs to inhibit [Ca^2+^]_i_ and relax ASM (by this mechanism), is dependent on which GPCR is acting to stimulate [Ca^2+^]_i_ and contract the airway. Thus in the current work we utilized multiple TAS2R agonists for the three highest expressing TAS2R subtypes in HASM, and agonists for multiple G_q_-and/or G_i_-coupled GPCRs which increase [Ca^2+^]_i_. We indeed demonstrate a dual mechanism for TAS2R signaling, where the inhibitory pathway hinges upon how [Ca^2+^]_i_ is increased in the ASM cell.

## Materials and Methods

### HASM Cells and Intact Bronchi

Primary HASM cultured cells were established from airways from deceased nonasthmatic individuals obtained from the National Disease Research Interchange (NDRI) (Philadelphia, PA, http://ndriresource.org) as described [23] and isolated as previously reported [27]. Cells were maintained in HAM’s F12 medium with 10% fetal bovine serum, 1% penicillin and streptomycin, 1% L-glutamine, 1.7 mM CaCl_2_, 12 mM NaOH, and 25 mM HEPES at 37°C in 95% air, 5% CO_2_. Cells were studied at passages 5–8. As previously noted, these cultures represent virtually 100% smooth muscle without epithelial or other cell types [28], and at these passage numbers the cells maintain pharmacologic and physiologic properties [22,29,30]. HASM cell viability after TAS2R agonist exposure was determined using the Vybrant and LIVE fluorescence assays (Life Technologies). Intact human bronchi were also obtained from NDRI and prepared as described [11]. The use of these tissues was in accordance with the guidelines of the Institutional Review Boards of the University of South Florida, Johns Hopkins University, and Thomas Jefferson University.

### HASM [Ca^2+^]_i_ Measurements

For measurement of [Ca^2+^]_i_ mobilization, we used the no-wash Fluo-4 Direct Calcium Assay kit (Life Technologies) according to the manufacturer’s instructions. Briefly, cells seeded in 96-well plates (40,000 cells/well) were loaded with the Ca^2+^ sensitive fluorescence indicator Fluo-4 and probenecid (2.5 mM) in Hank’s balanced salt solution containing (in mM), CaCl_2_ (1.3), MgCl_2_.6 H_2_O (0.5), MgSO_4_.6 H_2_O (0.4), KCl (5.3), KH_2_PO_4_ (0.4), NaHCO_3_ (4.2), NaCl (137.9), Na_2_HPO_4_ (0.3), D-Glucose (5.5), and HEPES (20). After 30 min incubation in the dark at 37°C under 5% CO_2_ / 95% air atmosphere followed by 30 min at 25°C in air and darkness, drugs were added, and the increase in [Ca^2+^]_i_ recorded over 120 sec (unless indicated otherwise) using the FlexStation3 plate reader (Molecular Devices). Fluorescence (excitation 485 nm, emission 525 nm, cut-off 515 nm) was measured every 1.52 sec. Baseline fluorescence background (F_o_) was captured for 16–19 sec before the addition of procontractile agonist plus or minus TAS2R agonists (50 μL, 5x each). When present, the final concentration of DMSO or DMF was below 0.25%, a concentration that did not affect the baseline signal. Calcium response (ΔF, arbitrary units) was calculated by subtracting basal fluorescence signal (F_o_, average of the first 10 readings) from the agonist peak value of fluorescence signal (F). The effect of co-stimulation with TAS2R agonists on [Ca^2+^]_i_ response evoked by procontractile agonists was expressed as percentage of ΔF in the presence (ΔF_a_) of the TAS2R agonist relative to the ΔF in the absence (ΔF_0_) of the TAS2R agonist [100 x (1- ΔF_a_ / ΔF_0_)].

### Cell Membrane Potential Assay

HASM cells plated in 96-well plates (40,000 cells/well) were studied using the FLIPR membrane potential dye BLUE (Molecular Devices). The sensitivity of this assay and comparison to patch-clamp recordings have been previously reported [31–33]. Cells were incubated with dye in Hanks’ balanced salt solution supplemented with 20 mM HEPES for 10 min at 25°C in the dark. Baseline fluorescence (excitation 530 nm, emission 565 nm, cut-off 550 nm) was measured for 16 sec before addition of agonists using the FlexStation3 instrument. Signals were acquired every 2 sec for 120 sec. Increase or decrease in fluorescence after cell stimulation with various agonists indicates cell membrane depolarization or hyperpolarization, respectively. Change in fluorescence is expressed as F—F_o_ (ΔF) as above.

### Magnetic Twisting Cytometry (MTC)

Dynamic changes in cell stiffness were measured in isolated HASM using forced motions of functionalized beads anchored to the cytoskeleton through cell surface integrin receptors, as described in detail previously [11,34]. The increase or decrease in cell stiffness is considered an index of smooth muscle contraction and relaxation, respectively, as has been previously described [34]. For each individual HASM cell, baseline stiffness was measured for the first 60 sec and after drug(s) addition, stiffness was measured continuously for the next 60 sec. For each cell, drug-induced changes in cell stiffness were normalized to its baseline stiffness prior to drug administration.

### Intact Airway Physiology

Third or fourth order bronchi from human lungs were dissected and cut into rings of 5 mm in length. They were studied in an isometric myograph (AD Instruments, Colorado Springs, CO) as previously described [11]. Briefly, rings were fitted between a fixed wire and a transducer-coupled wire in Krebs solution at 37°C bubbled with 95% O_2_ and 5% CO_2_. A passive tension of 5 mN was applied to the rings and tension recorded over the next 15 min to assure a stable baseline. Procontractile agonists were added to the bath at the indicted concentrations and measurements of force obtained until the maximal response was observed (typically 10 min). Then TAS2R agonists were added at the indicted concentrations, and tension measured over the next 10 min or until the maximal decrease in tension was observed.

### Drugs and Chemicals

Unless otherwise indicated, reagents were purchased from Sigma-Aldrich. Procontractile GPCR agonists used were histamine (3 μM), endothelin-1 (ET-1, 1 μM), bradykinin (BK, 5 nM), angiotensin II (Ang II, 100 μM), and acetylcholine (ACh, 1 mM). TAS2R agonists (1 nM to 2 mM) included aristolochic acid (AA), chloroquine (CQ), diphenhydramine (DPD), flufenamic acid (FFA), quinine (QUI), saccharin (SAC), strychnine (STRY), and yohimbine (YOH). Stock solutions were prepared in water or vehicle (DMSO or DMF) and diluted (5x final concentration) in calcium buffer or membrane potential buffer. Cell culture reagents including media, antibiotics, and fetal bovine serum were from Lonza and Life Technologies.

### Data Analysis

Results are expressed as the mean, standard error (SE) and the number of experiments (N). IC_50_ and EC_50_ values were obtained from concentration-effect non-linear regression sigmoid curves fitted using Prism (Graph Pad Software, La Jolla, CA). When biphasic curves were observed, values within the ascending portion were excluded, with the IC_50_ determined with data points within the descending portion of the curve. MTC results were analyzed using a nested effect method as described [35] using SAS V9.2 (SAS Institute Inc., Cary, NC). Statistical analyses for other studies consisted of student’s t-tests, performed with Prism and Excel. Two-tailed P values less than 0.05 were considered statistically significant. Figures were generated with Prism and Excel. In figures where gaps in the fitted line or axis appear, the lower concentration is baseline or “no drug”.

## Results

### TAS2R Agonists Stimulate [Ca^2+^]_i_ with Low Potency

The three highest expressing TAS2Rs in human ASM cells are TAS2R10, 14, and 31 [11]. We thus utilized CQ, YOH and STRY (TAS2R10), FFA, DPD (TAS2R14), AA and SAC (TAS2R 31), and QUI (TAS2R 10, 14, 31) for activating one or more of these three TAS2Rs. We used the Ca^2+^-sensitive fluorescent dye Fluo-4 to monitor changes in [Ca^2+^]_i_ in HASM cells. We first built upon our previous findings by determining the EC_50_ for each TAS2R agonist for stimulating [Ca^2+^]_i_ in HASM cells (S1 Table). These experiments were performed in the absence of co-incubation with procontractile agonists. As indicated, the EC_50_ values for these agonists for stimulating [Ca^2+^]_i_ in HASM are relatively high. However, these are consistent with results from [Ca^2+^]_i_ studies obtained using TAS2R cDNA-transfected cells [17] as well as taste bud cells [36].

### Chloroquine Inhibits the [Ca^2+^]_i_ Increase from Activation of Histamine Receptors in HASM Cells

**Fig 1:**
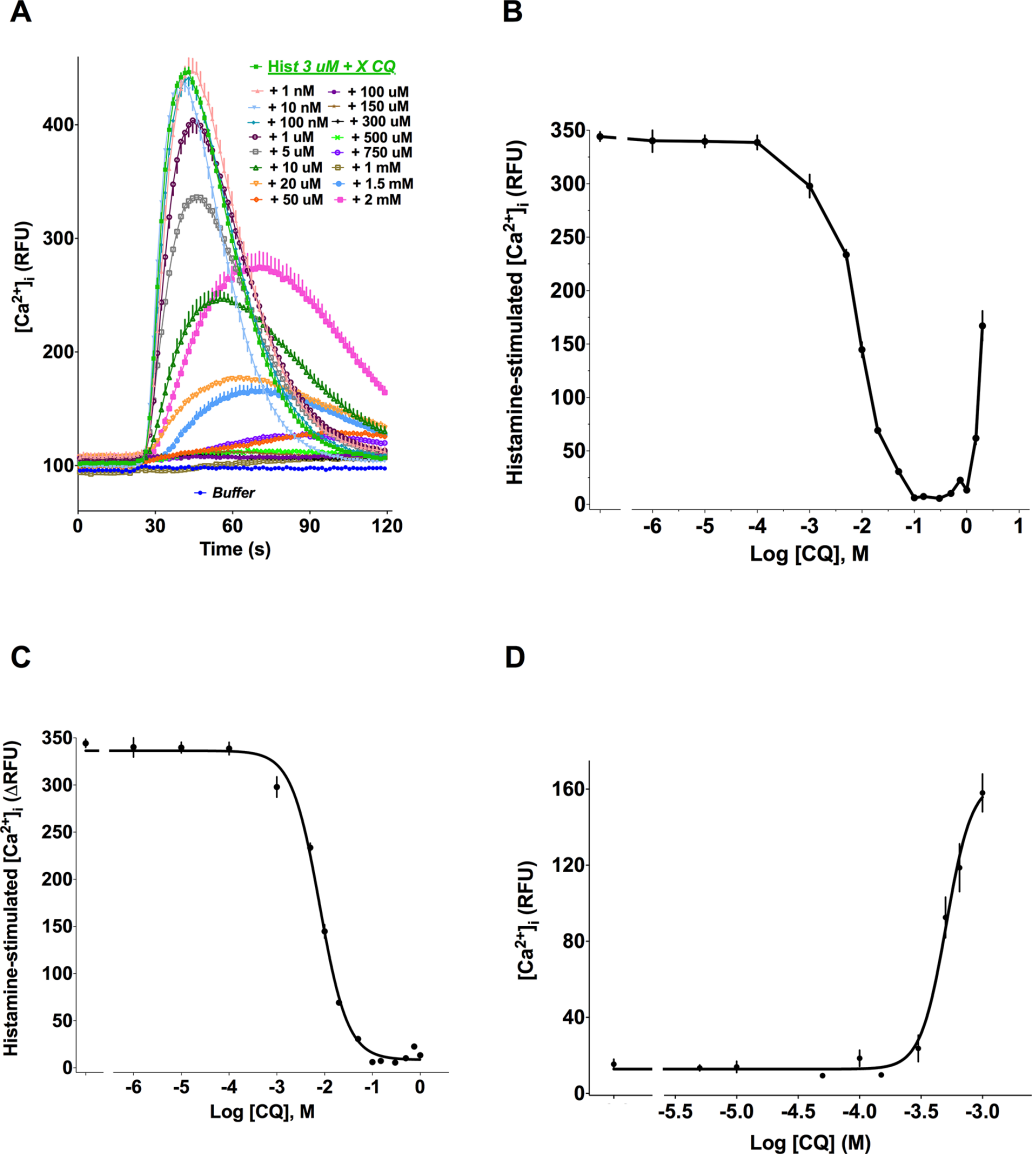
Biphasic effect of the TAS2R10 agonist CQ on HASM cell [Ca^2+^]_i_ mobilization. (A) Representative dose-response and time course of [Ca^2+^]_i_ in cells treated with 3 μM histamine and the indicated concentrations of CQ. (B) Biphasic [Ca^2+^]_i_ response to CQ in HASM concomitantly treated with 3 μM histamine. (C) Inhibitory limb of the [Ca^2+^] response to CQ in HASM concomitantly treated with 3 μM histamine. (D) Stimulation of [Ca^2+^] by CQ in HASM. There is no co-treatment with histamine in this experiment. Shown are representative results (mean ± SE of quadruplicates) of 3–5 independent experiments performed.

Stimulation of confluent HASM cells with histamine induced a rapid, dose-dependent, increase in [Ca^2+^]_i_, with an EC_50_ value of 0.6 ± 0.3 μM (not shown). Co-treatment of cells with 3 μM histamine and increasing concentrations of the TAS2R10 agonist CQ showed a dose-dependent *decrease* in the peak [Ca^2+^]_i_ beginning with 1 μM CQ and up to concentrations of ~1 mM CQ (Fig 1A). At higher concentrations of CQ, an increase in [Ca^2+^]_i_ was observed (Fig 1A). Thus a biphasic [Ca^2+^]_i_ response to CQ was evident in the presence of histamine stimulation (Fig 1B). Non-linear regression analyses of the inhibitory limb of the curve revealed an IC_50_ = 14.8 ± 5.8 μM (N = 4) (Fig 1C). Of note, CQ stimulates [Ca^2+^]_i_, in the absence of histamine with a EC_50_ of ~450 μM (Fig 1D and S1 Table). Together, these data indicate that CQ is more potent in inhibiting histamine-stimulated [Ca^2+^]_i_ than in *de novo* stimulation of [Ca^2+^]_i_ in HASM.

### Inhibition of [Ca^2+^]_i_ by TAS2R Agonists is Contingent Upon the Procontractile Receptor that Stimulates [Ca^2+^]_i_ Mobilization

**Fig 2:**
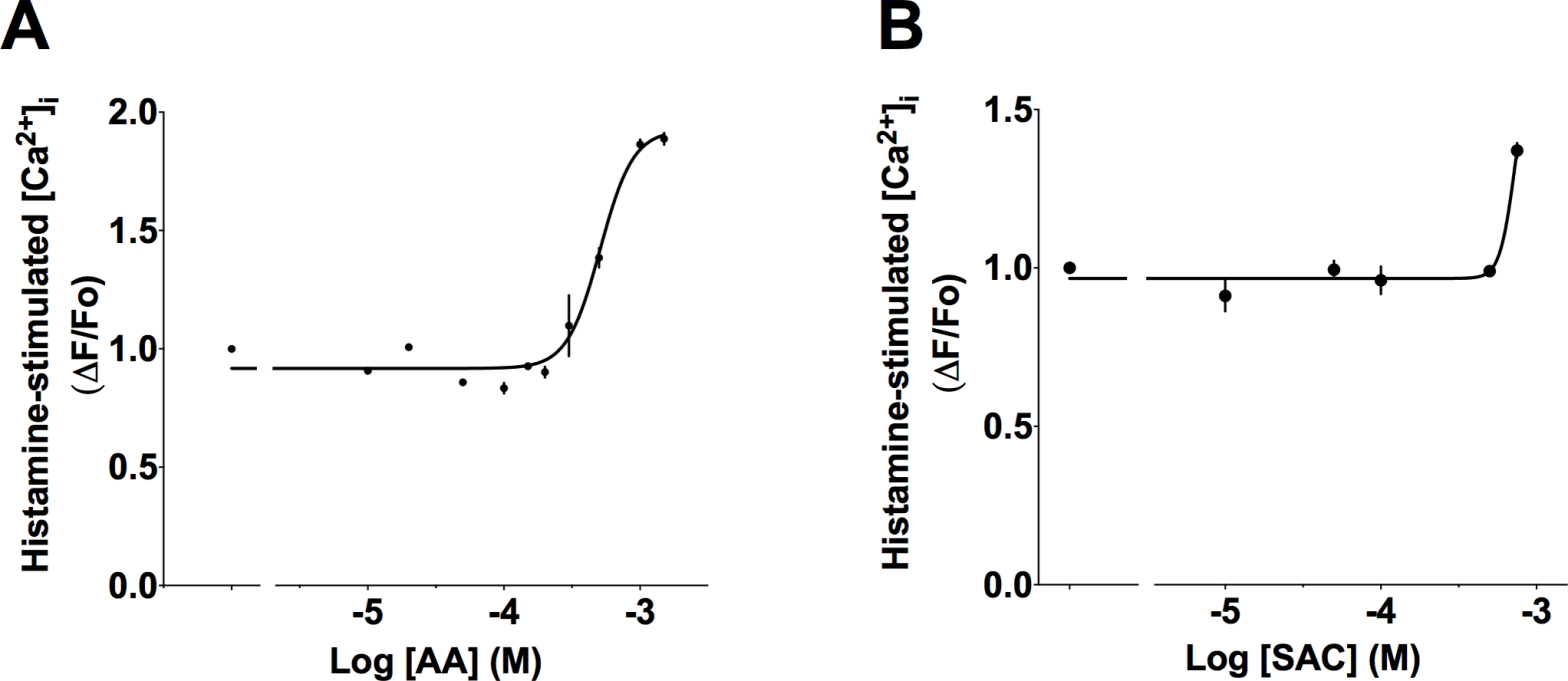
Effects of TAS2R31 agonists on histamine-stimulated [Ca^2+^] in HASM cells. Effect of varying concentrations of AA (A) or SAC (B) on 3 μM histamine-mediated [Ca^2+^]_i_ increase. Shown are representative results (mean ± SE of quadruplicates) of 3–5 independent experiments performed.

**Fig 3:**
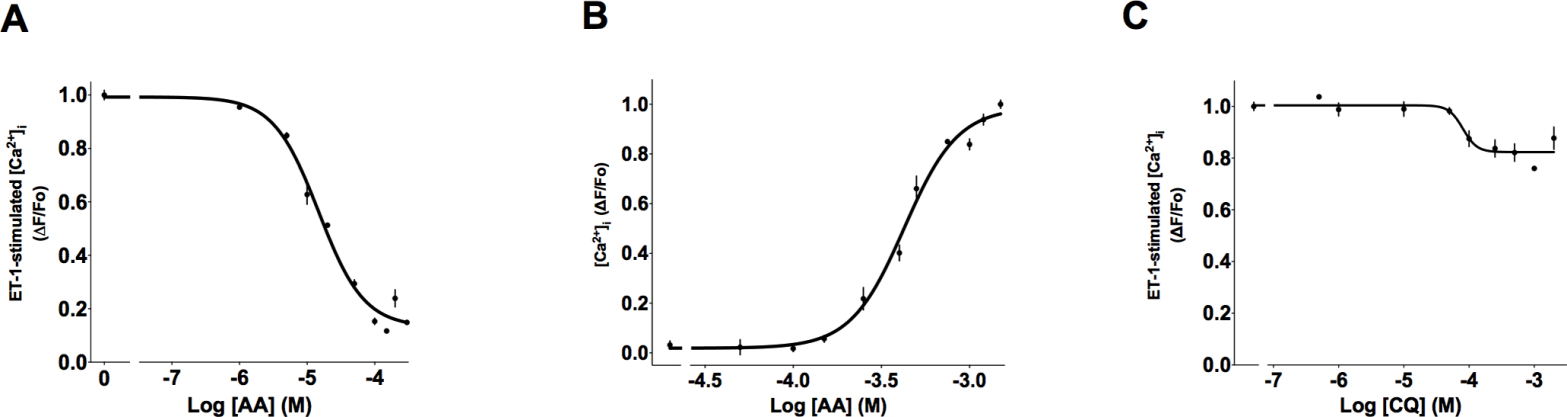
Inhibition of ET-1 stimulated [Ca^2+^]_i_ in HASM cells differs based on TAS2R agonist. (A) Inhibition of ET-1 stimulated [Ca^2+^] by AA. (B) AA stimulates [Ca^2+^]_i_ in the absence of ET-1. (C) CQ has a minimal inhibitory effect on ET-1 stimulated [Ca^2+^] (compared to histamine stimulated effect in Fig 1). Shown are representative results (mean ± SE of quadruplicates) of 3–5 independent experiments performed.

We next examined whether activation of TAS2R31 with AA would also inhibit [Ca^2+^]_i_ elicited by histamine. Fig 2A shows that in contrast to CQ, AA failed to decrease [Ca^2+^]_i_ evoked by histamine. The magnitude of the [Ca^2+^]_i_ peaks remained unchanged up to ~200 μM AA, above which an increase in the signal was detected. Similar to AA, co-treatment of HASM cells with SAC, a bitter tastant that activates TAS2R31, did not inhibit [Ca^2+^]_i_ stimulated by histamine (Fig 2B). The lack of inhibitory effect of AA and SAC on histamine-stimulated [Ca^2+^]_i_ prompted us to investigate whether AA inhibits elevated [Ca^2+^]_i_ induced by other GPCR agonists. Fig 3A shows that AA does indeed inhibit [Ca^2+^]_i_ that is elevated by ET-1. The calculated IC_50_ of AA with HASM cells stimulated with 1 μM ET-1 was 18.1 ± 6.7 μM (N = 3). As expected, AA in the absence of ET-1 stimulates [Ca^2+^]_i_ (Fig 3B), although this requires higher doses of AA to observe such stimulation. Remarkably, CQ, which was highly effective at inhibiting histamine-stimulated [Ca^2+^]_i_ (Fig 1C) showed a minimal effect on ET-1 stimulated [Ca^2+^]_i_ (Fig 3C). The inhibitory effect of the TAS2R agonists was not an artifact associated with drug-induced cell death (S1 Fig). Furthermore, this [Ca^2+^]_i_ inhibitory effect was fully reversed when the cells were washed to remove TAS2R agonist and rechallenged with procontractile agonist (S2 Fig). The efficacy of CQ to inhibit [Ca^2+^]_i_ elicited by histamine but not by ET-1, versus the efficacy of AA to inhibit [Ca^2+^]_i_ elicited by ET-1 but not by histamine, strongly suggests that the potential for TAS2R agonists to inhibit [Ca^2+^]_i_ is conditional upon the GPCR that stimulates [Ca^2+^]_i_ elevation.

**Fig 4:**
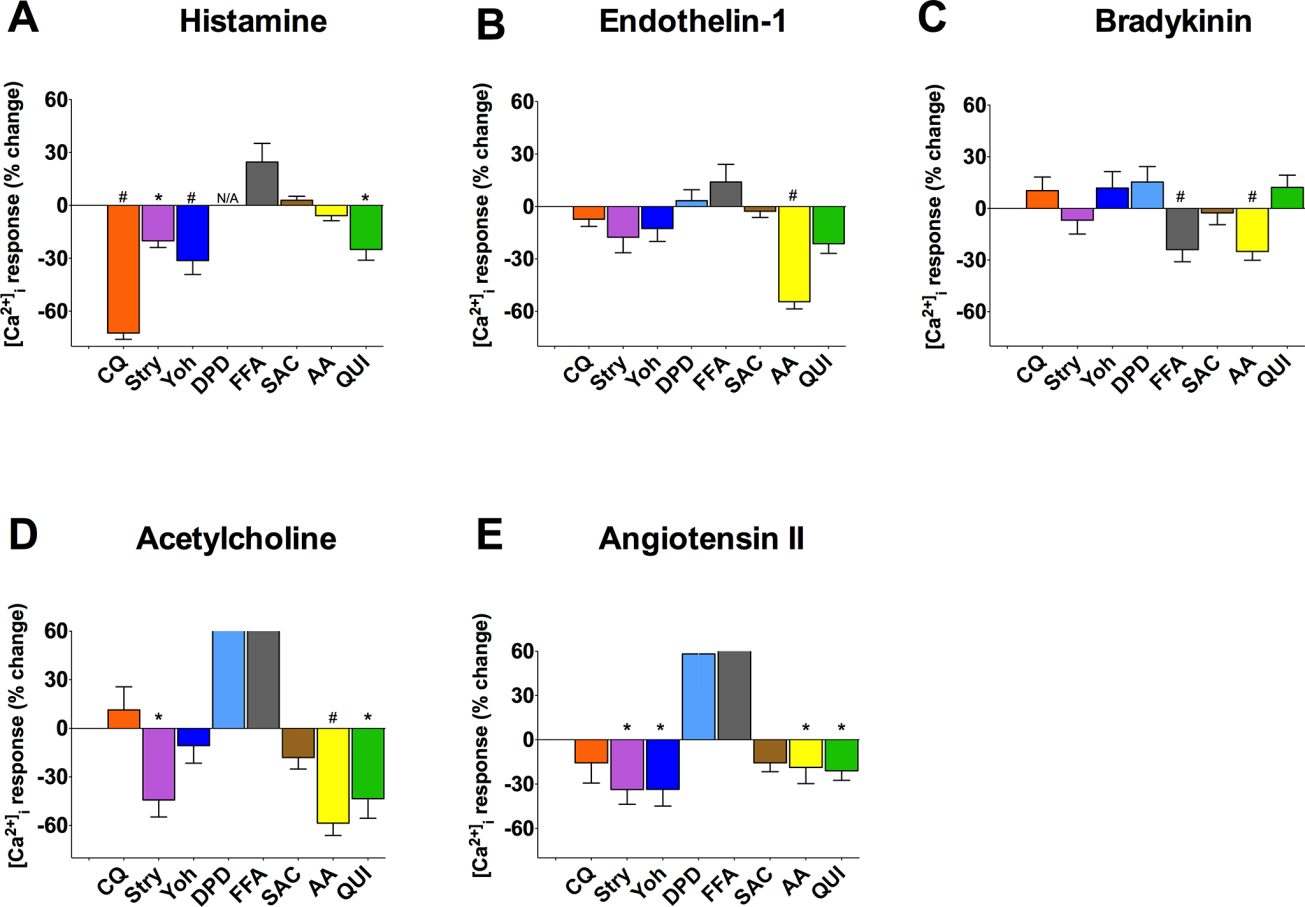
Quantitative effect of TAS2R agonists on [Ca^2+^]_i_ in HASM stimulated by five GPCR agonists. (A-E) Cells were treated with the 3 μM histamine, 1 μM ET-1, 5 mM BK, 1 mM ACh, or 100 μM Ang II in the absence (buffer) or presence of 50 μM of the indicated TAS2R agonists. Statistical analysis was not performed on co-treatments that resulted in [Ca^2+^]_i_ enhancement. Large positive values were truncated to allow visualization of the smaller values. *, P<0.05; # P<0.005 vs control (buffer). N = 5–8 experiments.

To better comprehend the extent of this selectivity, we utilized a battery of the aforementioned eight TAS2R agonists and five procontractile GPCR stimulators of [Ca^2+^]_i_: BK, ACh, Ang II, histamine, and ET-1. As introduced earlier, each of the TAS2R agonists stimulates [Ca^2+^]_i_ in HASM cells with low potency (S1 Table), and at the 50 μM concentration, no agonist consistently increased [Ca^2+^]_I_ levels above background. We therefore utilized 50 μM of TAS2R agonists in the screen. Because DPD is a histamine receptor antagonist, it was not studied in the context of histamine-stimulated [Ca^2+^]_i_ (denoted as N/A in figures). Quantitative analysis revealed that CQ, STRY, YOH and QUI inhibited [Ca^2+^]_i_ evoked by histamine while AA, FFA, and SAC had no effect (Fig 4A). On the other hand, AA and QUI suppressed [Ca^2+^]_i_ elicited by ET-1 but CQ, STRY, YOH, DPD and FFA did not. FFA and AA inhibited [Ca^2+^]_i_ stimulated by BK while AA, QUI, STRY, CQ, and YOH were without effect. Similar heterogeneity was observed when ACh and Ang II were utilized as the [Ca^2+^]_i_ stimulant (Fig 4D and 4E).

**Fig 5:**
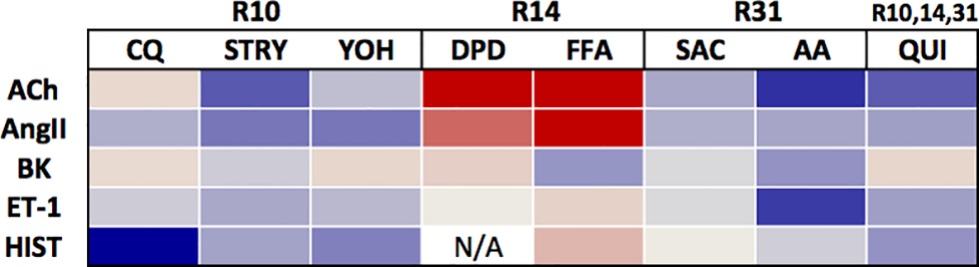
Heat map of the relative effects of TAS2R agonists on [Ca^2+^] stimulated by the indicated procontractile CPCR agonists. Data from the experiments in Fig 4 were normalized to maximal stimulation (red) or inhibition (blue). TAS2R agonists are on the top row and their subtype specificity shown. Procontractile GPCR agonists are listed in the first column. N/A, not applicable.

**Fig 6:**
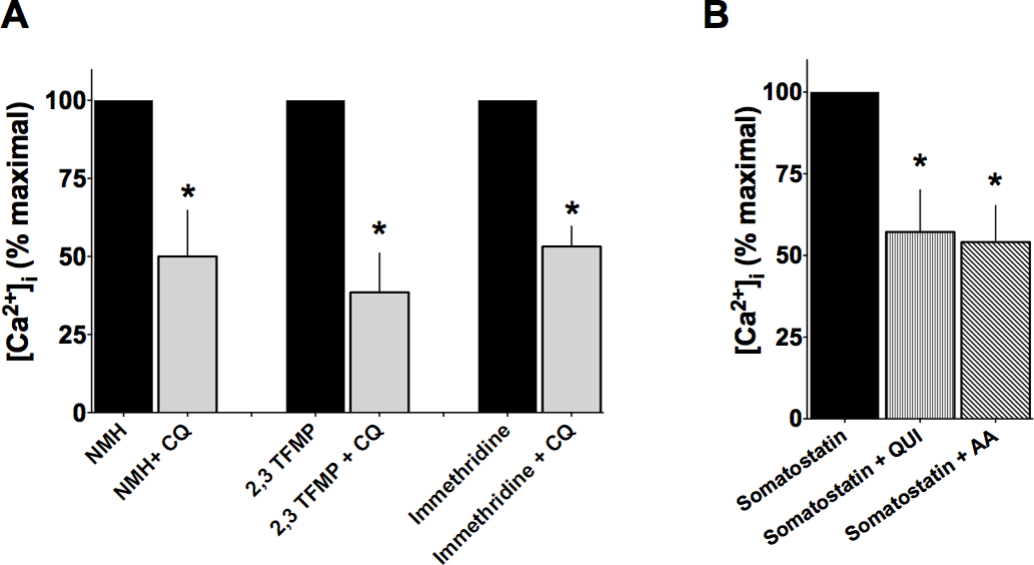
TAS2R inhibition of stimulated [Ca^2+^]_i_ is not dependent on G_i_ or G_q_ coupled receptor stimulation. (A) HASM were co-treated with G_q_-coupled HRH1 agonists NMH or 2,3 TFMP, or the G_i_-coupled HRH3 agonist immethridine and 50 μM CQ. (B) Somatostatin (G_i_-coupled) stimulated [Ca^2+^]_i_ is inhibited by QUI and AA. *, P<0.05, N = 4 experiments.

Fig 5 summarizes the above results for inhibition of [Ca^2+^]_i_ as a heat map, with TAS2R agonist subtype specificities noted for the different compounds. From this map, it does not appear that TAS2R inhibition of [Ca^2+^]_i_ stimulation by the various GPCR agonists is TAS2R subtype-dependent (note the lack of a pattern in the columns). We considered that the heterogeneity in the inhibitory response could be due to whether [Ca^2+^]_i_ was stimulated by a G_q_—vs a G_i_-coupled receptor. Of note, pertussis toxin treatment cannot be utilized for this differentiation, since it also inactivates the TAS2R G-protein gustducin [37]. We thus utilized subtype-specific agonists for the histamine HRH1 (G_q_-coupled) and HRH3 (G_i_-coupled) receptors in the absence or presence of CQ, and measured HASM [Ca^2+^]_i_ mobilization (Fig 6A). N-Methyl-histaprodifen (NMH, a HRH1 agonist) stimulated [Ca^2+^]_i_ was inhibited ~50% by CQ. Similarly, stimulation of HASM [Ca^2+^]_i_ by another HRH1 agonist, 2-((3-Trifluoromethyl)phenyl)histamine (2,3 TFMP), was also inhibited by CQ. Importantly, the G_i_-coupled HRH3 agonist immethridine-stimulated [Ca^2+^]_i_ was inhibited by CQ to a similar extent. To further assess the possibility of selectivity for G_q_- vs G_i_-coupled receptors for TAS2R-mediated inhibition, we exposed cells to 400 μM somatostatin, which activates the four somatostatin subtypes, all of which couple to G_i_ but not to G_q_. As shown in Fig 6B, [Ca^2+^]_i_ stimulation by somatostatin was also inhibited by TAS2R agonists to ~50%. Taken together, these data indicate that regardless of whether the GPCR couples to G_q_ or G_i_, TAS2R activation can reduce elevated [Ca^2+^]_i_, and thus the heterogeneity that we observe between contractile GPCR agonists and TAS2R agonists cannot be readily attributed to this mechanism.

### Heterogeneity in TAS2R Responses of HASM Cell Membrane Potential

**Fig 7:**
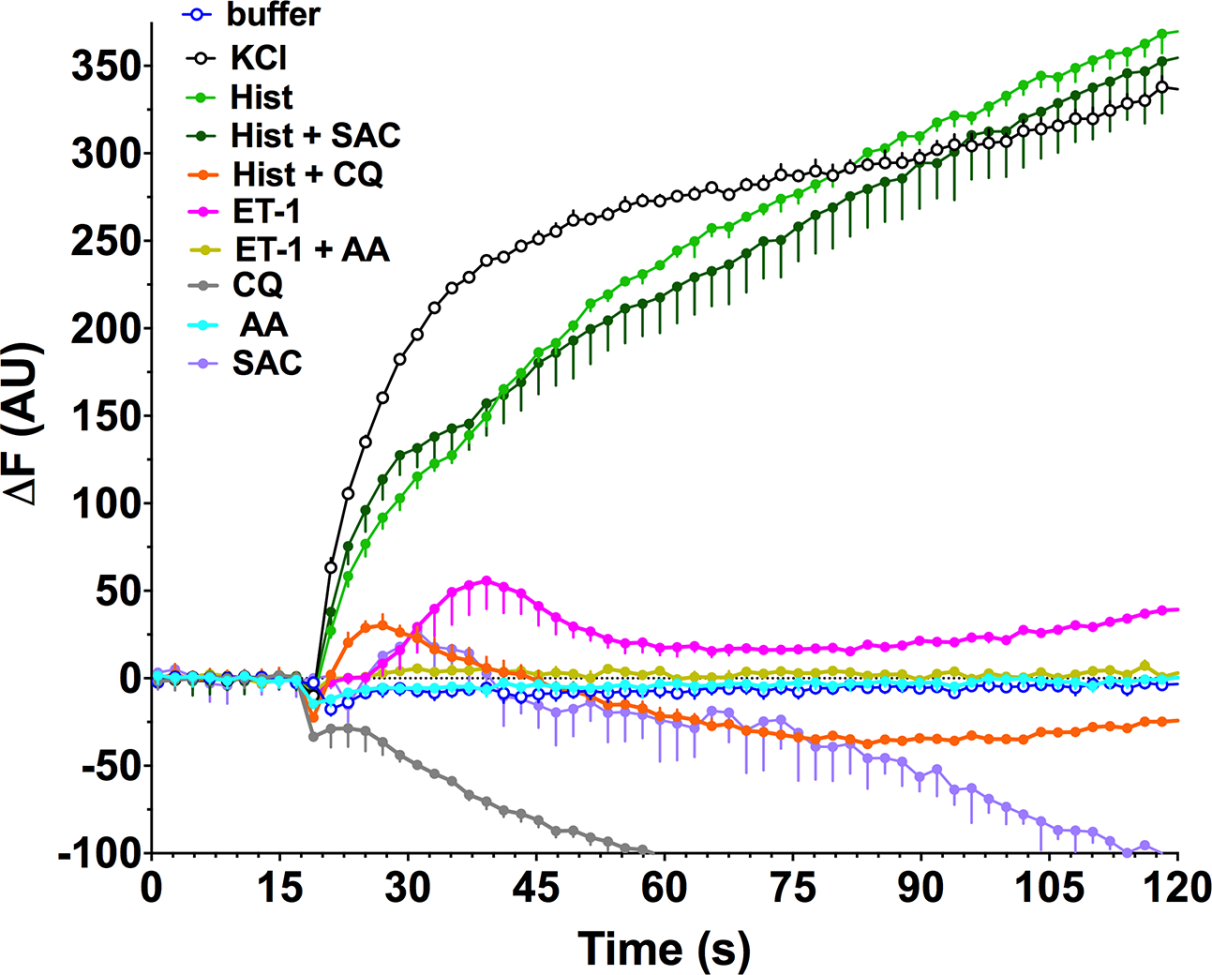
Heterogeneity of HASM membrane potential responses evoked by procontractile agonists co-treated with TAS2R agonists. Cultured HASM cells were studied using a fluorescence-based membrane potential dye. The indicated agents were added singly, or in combination, at the 16 sec time point. The final concentrations were: Hist 3 μM, CQ 50 μM, SAC 50 μM, ET-1 1 μM, AA 50 μM. Results are mean ± SE of triplicate determinations from a single representative experiment of 2–5 performed.

The increase in [Ca^2+^]_i_ induced by procontractile agonists promotes cell membrane depolarization and actin-myosin activation, ultimately leading to contraction. Conversely, hyperpolarization of the cell membrane antagonizes contraction. One of the proposed mechanisms by which TAS2R activation induces smooth muscle relaxation is a [Ca^2+^]_i_-dependent decrease in plasma membrane potential (hyperpolarization). We therefore explored the changes in membrane potential that occurred upon stimulation of HASM cells with bronchoconstrictive agonists in the presence and absence of co-administration of TAS2R agonists. We used a validated assay in which an increase or decrease in probe fluorescence is indicative, respectively, of depolarization or hyperpolarization of the cell membrane. Exposure of HASM to 60 mM KCl resulted in the expected depolarization (Fig 7). Similarly, the bronchoconstrictive GPCR agonist histamine caused a sustained depolarization. However, in the presence of 50 μM CQ, histamine-mediated membrane depolarization was markedly inhibited and indeed resulted in hyperpolarization. In contrast, SAC had no such effect. These results are consistent with the [Ca^2+^]_i_ inhibition studies, where CQ decreased histamine-stimulated [Ca^2+^]_i_ while SAC was ineffective (see Fig 4A). ET-1 also evoked depolarization, although it displayed an early peak with a lower-magnitude depolarization thereafter. Concomitant treatment with 50 μM AA blocked ET-1 depolarization (Fig 7), also consistent with the AA effect on lowering ET-1 stimulated [Ca^2+^]_i_ (see Fig 4B).

### The Dichotomy of CQ and AA Inhibition of [Ca^2+^]_i_ is Recapitulated in HASM Physiologic Responses

**Fig 8:**
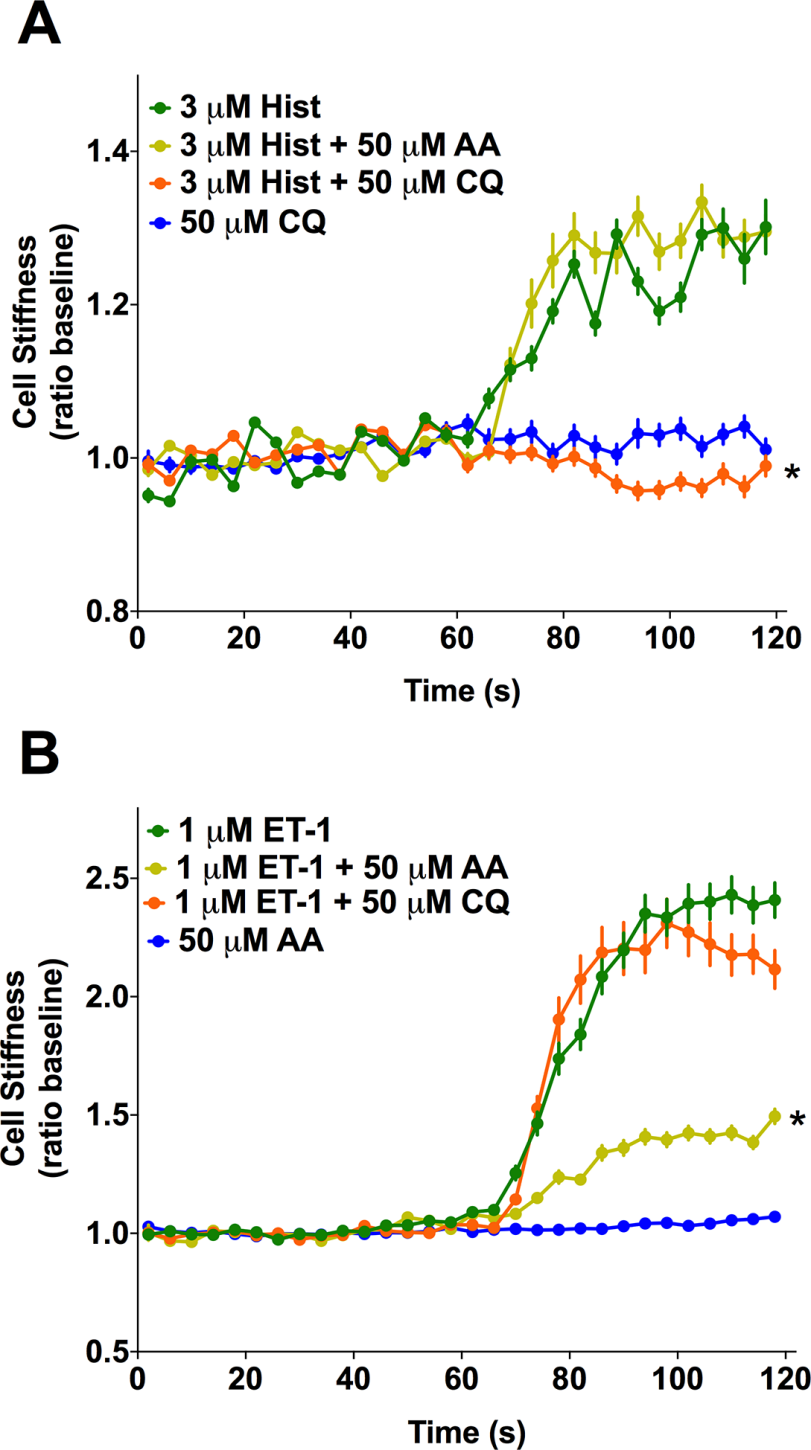
The heterogeneity of TAS2R inhibition of [Ca^2+^]_i_ is recapitulated in HASM physiological responses. (A, B) Isolated HASM in culture were studied using magnetic twisting cytometry. Cells were treated with 3 μM histamine or 1 μM ET-1 alone or together with the indicated TAS2R agonists (50 μM). * P<0.01 vs control (histamine or ET-1 with buffer). N = 303–400 cells per condition.

The above results suggest that HASM relaxation by a given TAS2R agonist would be dependent upon which GPCR is acting to contract the muscle. To test this, we utilized MTC, a sensitive method that detects changes in stiffness in single cells and is considered a surrogate for ASM contraction [34]. As shown in Fig 4A and 4B, a clear dichotomy in TAS2R responses is observed between histamine and ET-1 stimulated [Ca^2+^]_i_. CQ inhibits the former but not the latter, which is in contrast to that of AA, which inhibits ET-1-but not histamine-stimulated [Ca^2+^]_i_. To ascertain if these biochemical findings correlate with the expected physiologic effects, HASM cells were treated with 3 μM histamine alone, or in combination with 50 μM of either AA or CQ. Similarly, HASM cells were treated with 1 μM ET-1 alone, or in combination with 50 μM of either AA or CQ. HASM cell stiffness was measured for 60 sec and normalized to baseline (absence of any drugs). As shown in Fig 8A, histamine caused the expected increase in cell stiffness. Co-administration with 50 μM CQ, which inhibited histamine-evoked [Ca^2+^]_i_ by ~70%, fully blocked the histamine-mediated increase in cell stiffness (Fig 8A). In contrast, and consistent with the [Ca^2+^]_i_ results of Fig 4A, AA had no effect on histamine-induced cell stiffening. For ET-1 evoked stiffness (Fig 8B), AA caused an attenuation of the stiffness response, amounting to a ~60% reduction. The magnitude of this response is nearly identical to the reduction in [Ca^2+^]_i_ shown in Fig 4B. Consistent with the lack of an inhibition of the [Ca^2+^]_i_ response to ET-1, CQ had no physiological effect on the HASM stiffness response to ET-1 (Fig 8B).

**Fig 9:**
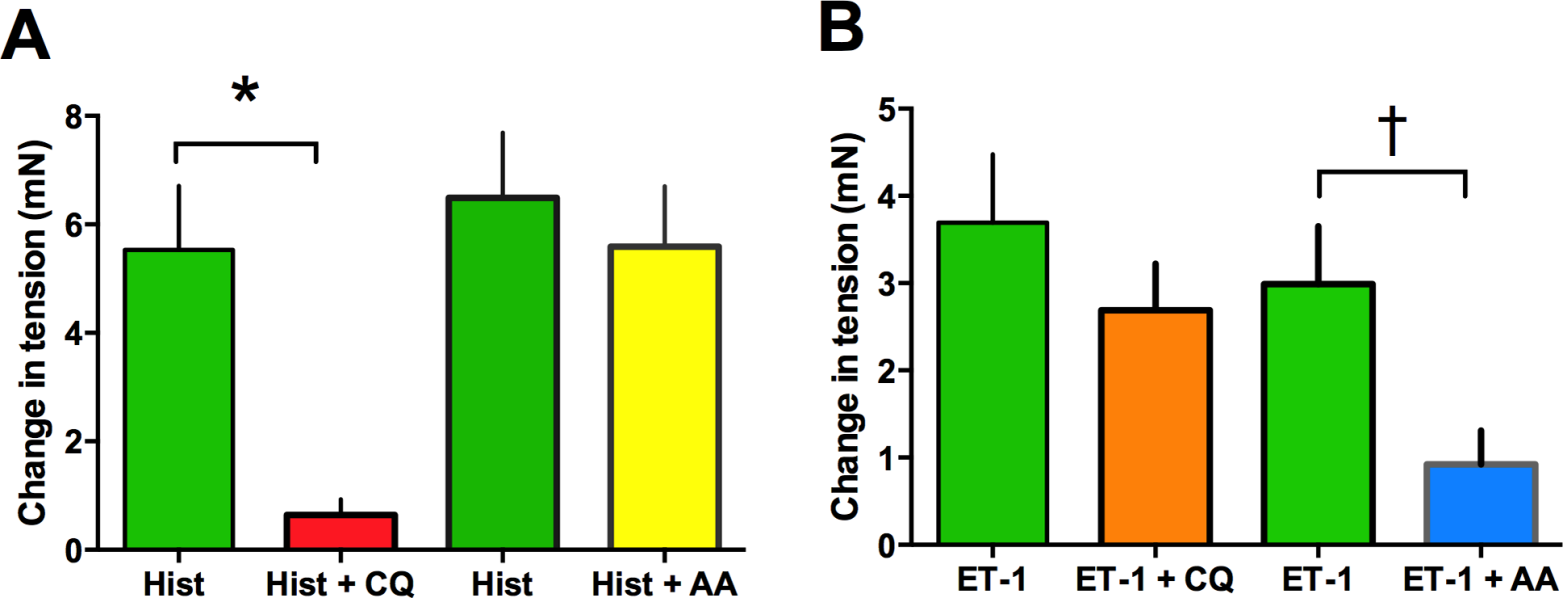
TAS2R inhibition of contracted human bronchi. Results are from experiments performed on 10–15 airway rings from three different donated lungs. The concentrations of drugs were: histamine 10 μM, ET-1 1 μM, AA 100 μM, CQ 100 μM. *, P<0.001; † P<0.05, compared to histamine or ET-1 alone.

To confirm these results from isolated cell mechanics experiments, we studied human bronchi in the *ex vivo* setting, measuring contraction and relaxation of airway rings in a lateral myograph. In this system, the coordinated effect of the ASM cell phenotypes can be ascertained in the context of the intact airway. Mean data from measurements using 10–15 rings derived from three independent donors are shown in Fig 9. When airways were contracted with histamine, CQ evoked ~90% relaxation. However, AA had no effect on histamine-mediated tension (Fig 9A). In contrast, when airways were contracted with ET-1, AA caused ~75% relaxation while CQ had a non-significant effect (Fig 9B). These results are fully consistent with the results from the MTC experiments with isolated ASM cells, as well as the membrane potential and [Ca^2+^]_i_ inhibition results.

## Discussion

As introduced earlier, TAS2Rs expressed on HASM cells represent a novel target for a new class of direct bronchodilators for the treatment of obstructive lung diseases such as asthma and chronic obstructive lung disease. Here we report the function of eight TAS2R agonists acting on HASM under physiologically relevant conditions of exposure to procontractile GPCR agonists. We hypothesized that TAS2R activation decrease [Ca^2+^]_i_ stimulated by these procontractile GPCR agonists, and, that this inhibitory effect occurs with lower concentrations of TAS2R agonists than those that promote the TAS2R-mediated [Ca^2+^]_i_ stimulatory pathway. We found that the efficacy of TAS2R agonists to oppose the increase in [Ca^2+^]_I_ evoked by other GPCRs is contingent upon which procontractile GPCR is being activated and that both G_i_ and G_q_ coupled receptors are subject to TAS2R inhibition. Furthermore, the decrease in [Ca^2+^]_i_ by TAS2R agonists was associated with a decrease in cell membrane depolarization. When a given TAS2R agonist-GPCR procontractile agonist pair did not reveal a TAS2R-mediated inhibition of [Ca^2+^], there was consistently no effect on membrane potential. The physiological consequences of these TAS2R-mediated events were ascertained by measuring HASM cell mechanics (which examines the effect on isolated smooth muscle cells) and by measuring force in intact human bronchi (which ascertains the effect on coordinated smooth muscle function of the intact airway). The heterogeneity observed for certain pairs for inhibiting [Ca^2+^]_i_ was indeed recapitulated in measurements of stiffness in single cells. Cell stiffness, which was increased by the procontractile agonists was antagonized only by TAS2R agonists that decreased [Ca^2+^] (and opposed membrane depolarization) under the same conditions. Finally, intact human airway responses were consistent with the results from the [Ca^2+^]_i_, membrane potential, and isolated cell mechanics studies. Taken together, these findings demonstrate the relevance of this TAS2R pathway to human physiologic responses.

Our initial identification and characterization of TAS2Rs on mouse and human ASM centered around the increase in [Ca^2+^]_i_ as a key intracellular event leading to ASM relaxation [11]. Indeed, agonists for these receptors caused robust increases in [Ca^2+^]_i_ in isolated HASM cells, that was blocked by G_βγ_ and PLC inhibitors, and substantially attenuated by an IP_3_ receptor antagonist. In addition, human and mouse ASM have been shown to express gustducin [11,15]. This stimulatory pathway pointed towards a taste cell-like response, where the G_βγ_ activation of PLC caused IP_3_ production which activated the endoplasmic reticulum IP_3_ receptor, releasing [Ca^2+^] from these stores into an intracellular space. However, we found that the temporal and spatial distribution of this Ca^2+^ in HASM cells was suggestive of the activation of one or more cell surface channels such as the large conductance calcium dependent K^+^ channel (BK_Ca_), which leads to ASM cell hyperpolarization. There appears to be additional mechanisms by which this specialized pool of [Ca^2+^]_i_ evoked from TAS2R activation leads to relaxation [15]. Regardless of potential mechanism, TAS2R agonists that fail to increase HASM [Ca^2+^]_i_ also fail to relax [11]. This stimulatory pathway appears to be somewhat inefficient, in that most TAS2R agonists activate [Ca^2+^]_i_ with EC_50_ values in the high μM to mM range in HASM (S1 Table) and in transfected cell lines [17]. With this current report and studies by others using different cells [15], it is now apparent that TAS2Rs acting through a distinct pathway can also *decrease* [Ca^2+^]_i_ in HASM cells that have been stimulated by other means. This decrease in [Ca^2+^]_i_ would be expected to cause relaxation since it antagonizes the procontractile GPCR-mediated elevated [Ca^2+^]_i_, and thus supports the concept that TAS2Rs in ASM cells initiate two signaling events. The lower efficiency transduction pathway results in an increase in [Ca^2+^]_i_ and subsequent membrane hyperpolarization and relaxation. The more efficient process, instead, acts to functionally compete with a stimulated increase in [Ca^2+^]_i_ and dampen, or prevent, depolarization. The physiologic response, then, is relaxation from the contracted state. We also show that there is heterogeneity of the response that hinges upon which procontractile GPCR is being stimulated.

**Fig 10:**
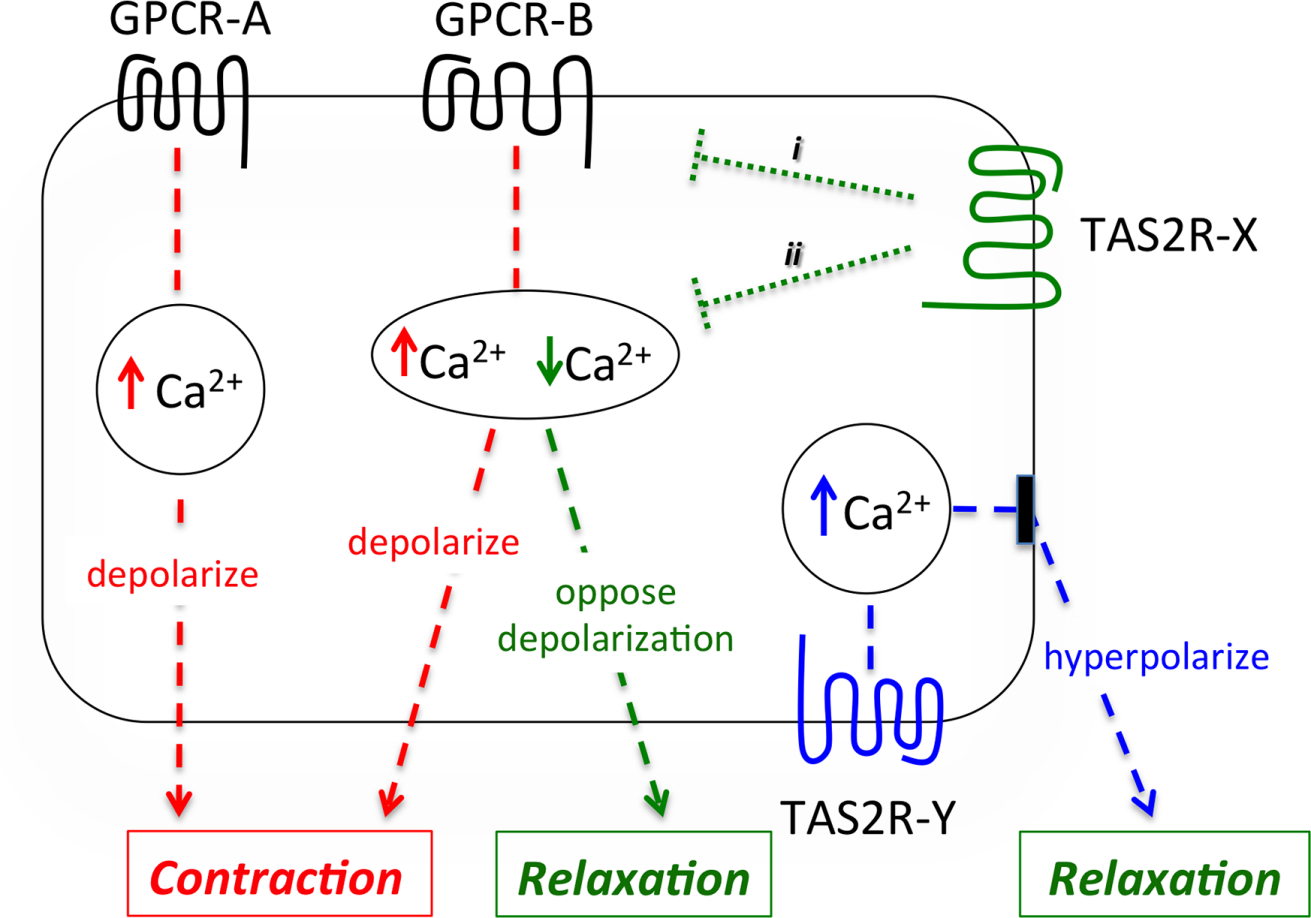
Model of TAS2R signaling to relaxation of HASM. Stimulation of GPCR-A results in an increase in [Ca^2+^]_i_, depolarization, and contraction (red). GPCR-A is not influenced by TAS2R interaction, thus [Ca^2+^]_i_ mobilization and ultimately contraction is not affected. Activation of certain GPCRs (GPCR-B) also promote contraction by increasing [Ca^2+^]_i_, but are influenced (green dotted lines) by TAS2R agonists (acting at TAS2R-X). This results in a decrease in [Ca^2+^]_i_, which opposes depolarization and relaxes HASM (green). This GPCR-B/TAS2R-X interaction could be in proximal (i) components or other downstream components (ii). TAS2R-Y does not specifically interact with a procontractile GPCR, but relaxes HASM by a calcium-dependent transducer, such as BK_Ca_, that hyperpolarizes the membrane (blue).

We propose the operational model depicted in Fig 10, where three [Ca^2+^]_i_ pools are indicated. GPCR-A is a bronchoconstrictive receptor whose activation elevates [Ca^2+^]_i_ leading to contraction. Because there is no interaction with TAS2R-X signaling, agonists for this TAS2R do not inhibit [Ca^2+^]_i_ or reverse depolarization from this bronchoconstrictive receptor and thus we would expect no TAS2R physiological effect. An example of this signaling is histamine (representing GPCR-A) in the presence of AA, which causes no change in [Ca^2+^]_i_ or relaxation (Figs 4A, 8A and 9, respectively). In contrast, the signaling of GPCR-B to elevate [Ca^2+^]_i_ interacts with the signaling of TAS2R-X. This could occur in a very early event (Fig 10, i) such as through βγ as has been suggested [15], or at later points (Fig 10, ii) such as between [Ca^2+^]_i_ pools. In this case, TAS2R-X inhibits the [Ca^2+^]_i_ elevated by GPCR-B, leading to reversal of depolarization and relaxation. An example of this scenario is AA acting at TAS2R31 to inhibit endothelin receptor-stimulated [Ca^2+^] (Fig 4A), reverse depolarization (Fig 7), decrease cell stiffness (Fig 8A), and relax intact airways (Fig 9B). A third scenario in the model is the action of TAS2R-Y in the absence of [Ca^2+^] stimulation by G_q_- or G_i_-coupled GPCRs. Here, there is no interaction between the signaling of the TAS2R and the bronchoconstrictive GPCR. In this instance, TAS2R-Y relaxes the ASM cell by a [Ca^2+^]_i_-dependent cell surface transducer, which hyperpolarizes the membrane when [Ca^2+^]_i_ is increased by TAS2R-Y agonists, acting to decrease ASM tone. This represents the less efficient pathway, based on EC_50_ values being higher than those for [Ca^2+^]_i_ inhibition. An example of this scenario is SAC, which clearly stimulates [Ca^2+^]_i_, hyperpolarizes the HASM cell membrane, and relaxes HASM in the absence of a contractile stimulus [11].

In humans, there are 25 TAS2R subtypes expressed on taste buds, which presumably evolved to trigger avoidance of ingestion of toxic bitter plants [17,18]. Most TAS2R subtypes are activated by large numbers of naturally occurring bitter substances, and given that these substances come into direct contact with the tongue, probably evolved towards low affinity and broad ligand recognition to accomplish this function. Recently, TAS2Rs have been identified on other cell types and regions of the body, including the nose, gastrointestinal tract, thyroid, lung, heart, lymphocyte, brain and testes. This suggests a previously unrecognized chemosensory system in the body that may respond to exogenous substances ingested in food or endogenously produced substances such as from resident bacteria [38,39]. These extraoral receptors may represent targets for new therapeutics, such as the TAS2R subtypes expressed on ASM which act to markedly relax the muscle resulting in bronchodilation. The dual pathway that we show for TAS2Rs in HASM cells offers intriguing possibilities for drug discovery and design. In the initial phase, measurement of fluorescent-based [Ca^2+^] measurements using cells transfected with the cDNA for a specific TAS2R subtype will provide a platform for compound screening. Typically, the cDNA for a Ggust/G44 chimeric G-protein is also transfected, which directs signaling to PLC activation and an increase in [Ca^2+^]_i_ [17]. In this system, [Ca^2+^]_i_ acts as a readily acquired indicator of receptor activation, but is not necessarily the physiologically relevant signal. The “inhibition pathway”, therefore, is not revealed in this screening approach. In the second phase, moving lead compounds from the transfected cell studies to HASM cell-based assays would be appropriate, measuring both TAS2R-stimulation of [Ca^2+^]_i_ as well as TAS2R mediated inhibition of [Ca^2+^]_i_ stimulated by a procontractile GPCR agonist. Our findings caution against excluding compounds based on the lack of inhibition of [Ca^2+^]_i_ elevated by a single GPCR procontractile agonist (Fig 4). Rather, multiple such contractile agonists would need to be explored. This would also be the case for membrane potential assays as well as physiological assays.

There is considerable precedence for receptors within the GPCR superfamily (of which the TAS2R family is a member) coupling to multiple pathways, including those with competing or opposing effects. For example, the α_2_ARs couple to G_αi_ (inhibiting cAMP) and to G_αs_ (stimulating cAMP) [40]. The latter function requires higher doses of agonist compared to the inhibitory function due to the lower efficiency of coupling [40,41]. Specific regions within the intracellular loops of the α_2A_AR have been identified which direct coupling to G_αs_ or G_αi_ [42,43], thus showing receptor structure as the basis for these multifunctional events. In this instance one receptor couples to two G-proteins with opposing actions on the effector adenylyl cyclase. Multifunctional signaling can also be from non-G-protein interactions. For example, activation of the angiotensin II type 1A receptor couples to PLC activation via G_q_, but also activates c-Jun amino-terminal Kinase 3 (JNK3) by βarrestin-2 binding to the receptor, which provides a scaffold for JNK3 activation [44]. GPCR signaling can also be directed based on spatial distribution of the receptor, G-protein, or effector within the cell, thereby resulting in specialized pools of second messenger [11,45,46]. These and other mechanisms of multifunctional GPCR signaling have been reviewed elsewhere [47–49]. The mechanisms by which TAS2Rs couple to both [Ca^2+^]_i_ stimulation and inhibition are not readily apparent. This phenomenon is made even more complex by the heterogeneity of the inhibitory response, which is dependent upon which GPCR is providing the [Ca^2+^]_i_ stimulation.

In conclusion, TAS2Rs on HASM are recognized to stimulate [Ca^2+^] with high agonist concentrations which results in membrane hyperpolarization and HASM relaxation. In addition, when TAS2Rs are activated by lower concentrations of agonist under conditions of elevated [Ca^2+^] by various bronchoconstrictors, they inhibit this [Ca^2+^]_i_ increase, oppose membrane depolarization, and thus relax precontracted HASM. The inhibitory response is dependent on which GPCR is acting to stimulate [Ca^2+^] mobilization. This suggests a compartmentalization of [Ca^2+^]_i_ signals in HASM, of which some are accessible to TAS2Rs, or, other forms of signaling interactions between TAS2Rs and bronchoconstrictive receptors.

## Supporting Information

S1 Table EC_50_ Values of TAS2R Agonists for Stimulating [Ca^2+^]_i_ in HASM Cells. (PDF)

S1 Fig The inhibitory effect of TAS2R agonists on [Ca^2+^] signaling is not accompanied by cell death. A) Cell death was determined using the Vybrant assay (Life Technologies), which quantitates the formation of reduced red fluorescent resazurin from a coupled enzymatic reaction in which NADPH is generated from the activity of glucose-6-phosphate dehydrogenase released from dying cells. 40,000 HASM cells/well were treated with buffer or buffer with 50 μM or 1 mM of the indicated TAS2R agonists for 5 min. As a positive control, cells were treated with lysis buffer. Data is from 4–7 experiments performed in triplicate. P>0.05 for all agonists compared to buffer, indicating no significant cell death. B) The proportion of live HASM cells was determined with the LIVE assay (Life Technologies) which measures intracellular esterase activity on calcein-AM which fluoresces green when hydrolyzed. Cells were plated at 40,000/well and treatments were with buffer alone or buffer with 50 μM of the indicted TAS2R agonists for 5 min. No agonist caused a decrease in viable cells. Data is from 4–6 experiments performed in triplicate. P = 0.04 for YOH which was greater than control (buffer).
(TIFF)

S2 Fig The inhibitory effect on [Ca^2+^] mobilization of selected TAS2R agonists is reversible. 40,000 HASM cells/well were exposed to buffer alone (representing “untreated”) or buffer with 50 μM CQ or AA for 5 min. Cells were washed twice with PBS, and then [Ca^2+^]_i_ mobilization measured in response to 3 μM histamine, 1 μM ET-1, or 1 μM ionomycin. The responses to histamine and ET-1 (as well as ionomycin) were no different in cells pretreated with CQ or AA, compared to pretreatment with buffer alone, indicated a reversal of TAS2R agonist effect. Data is from 4–5 experiments performed in triplicate.
(TIFF)
