# Supplementary material for: Pleiotropic Effects of Bitter Taste Receptors on [Ca2+]i Mobilization, Hyperpolarization, and Relaxation of Human Airway Smooth Muscle Cells
Source: PLoS One. 2015 Jun 29;10(6):e0131582. doi: 10.1371/journal.pone.0131582 (PMC4485472; doi:10.1371/journal.pone.0131582)
Supplement: S1 Table — (PDF) [file pone.0131582.s001.pdf]

Table S1 EC_{50} Values of TAS2R Agonists for Stimulating $[Ca^{2+}]_i$ in HASM Cells.

Compound	EC50 (μM) mean ± SE
aristolochic Acid	333.1 ± 29.8
chloroquine	452.8 ± 66.0
diphenhydramine	185.5 ± 78.3
flufenamic Acid	137.6 ± 76.3
quinine	666.7 ± 42.9
saccharin	558.2 ± 62.3
strychnine	>2mM
yohimbine	>2mM

Results are from at least four experiments, except for strychnine (n=2).
