# Supplementary figures and images for: Pleiotropic Effects of Bitter Taste Receptors on [Ca2+]i Mobilization, Hyperpolarization, and Relaxation of Human Airway Smooth Muscle Cells
Source: PLoS One. 2015 Jun 29;10(6):e0131582. doi: 10.1371/journal.pone.0131582 (PMC4485472; doi:10.1371/journal.pone.0131582)

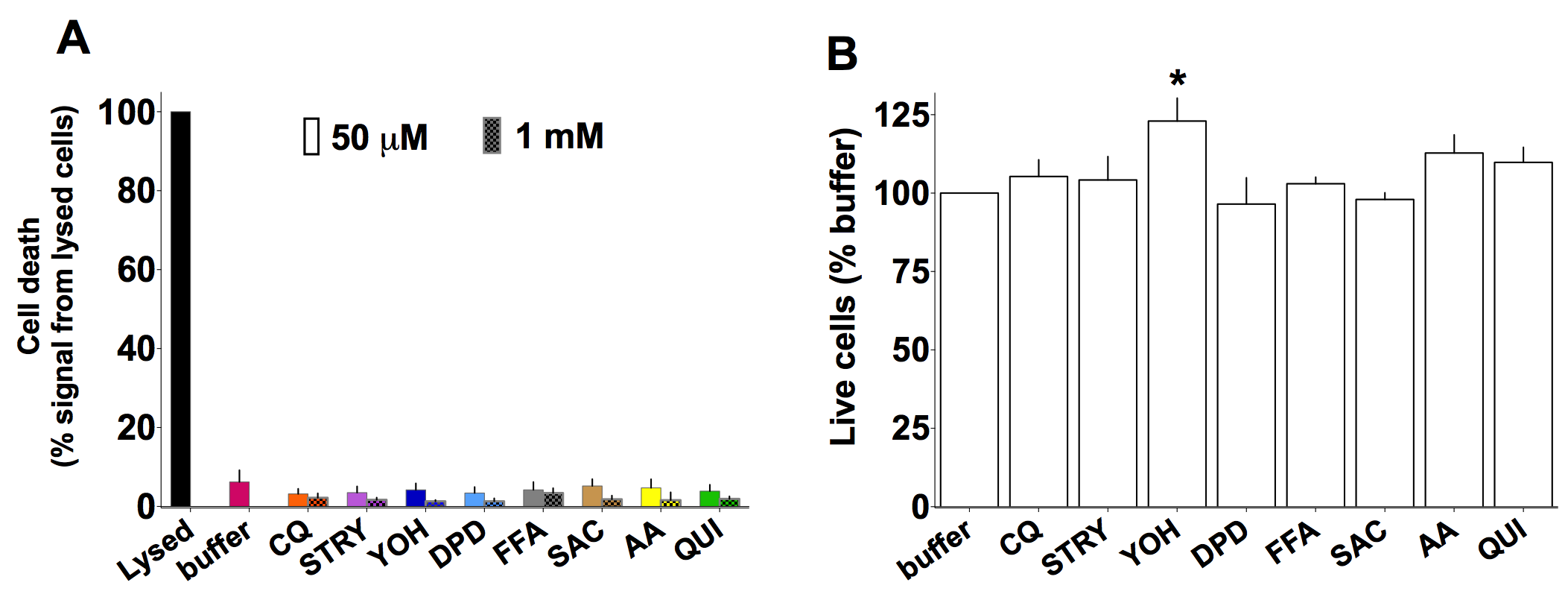

Supplement: S1 Fig — A) Cell death was determined using the Vybrant assay (Life Technologies), which quantitates the formation of reduced red fluorescent resazurin from a coupled enzymatic reaction in which NADPH is generated from the activity of glucose-6-phosphate dehydrogenase released from dying cells. 40,000 HASM cells/well were treated with buffer or buffer with 50 μM or 1 mM of the indicated TAS2R agonists for 5 min. As a positive control, cells were treated with lysis buffer. Data is from 4–7 experiments performed in triplicate. P>0.05 for all agonists compared to buffer, indicating no significant cell death. B) The proportion of live HASM cells was determined with the LIVE assay (Life Technologies) which measures intracellular esterase activity on calcein-AM which fluoresces green when hydrolyzed. Cells were plated at 40,000/well and treatments were with buffer alone or buffer with 50 μM of the indicted TAS2R agonists for 5 min. No agonist caused a decrease in viable cells. Data is from 4–6 experiments performed in triplicate. P = 0.04 for YOH which was greater than control (buffer). (TIFF) [file pone.0131582.s002.tiff]

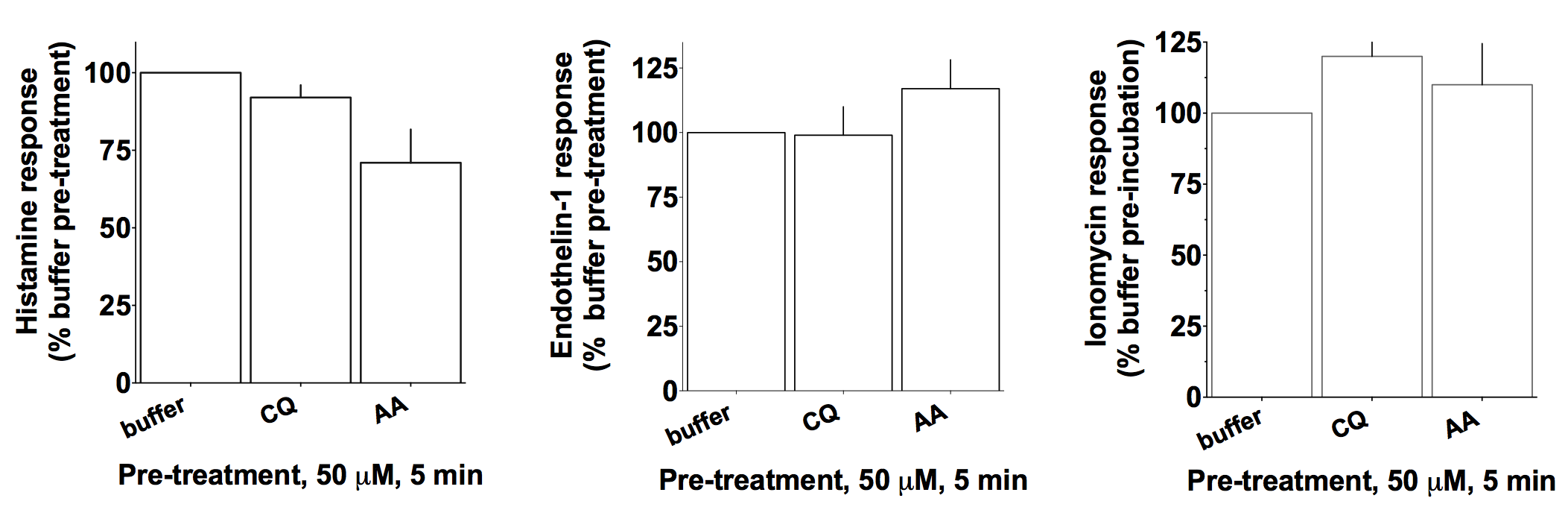

Supplement: S2 Fig — 40,000 HASM cells/well were exposed to buffer alone (representing “untreated”) or buffer with 50 μM CQ or AA for 5 min. Cells were washed twice with PBS, and then [Ca2+]i mobilization measured in response to 3 μM histamine, 1 μM ET-1, or 1 μM ionomycin. The responses to histamine and ET-1 (as well as ionomycin) were no different in cells pretreated with CQ or AA, compared to pretreatment with buffer alone, indicated a reversal of TAS2R agonist effect. Data is from 4–5 experiments performed in triplicate. (TIFF) [file pone.0131582.s003.tiff]
